# Genomic Analysis of ATP Efflux in *Saccharomyces cerevisiae*

**DOI:** 10.1534/g3.115.023267

**Published:** 2015-11-18

**Authors:** Theodore W. Peters, Aaron W. Miller, Cendrine Tourette, Hannah Agren, Alan Hubbard, Robert E. Hughes

**Affiliations:** * The Buck Institute for Research on Aging, Novato, California 94945; † School of Public Health, Division of Biostatistics, University of California, Berkeley, California 94729-7358

**Keywords:** yeast, ATP, secretion, mitochondria, TORC1

## Abstract

Adenosine triphosphate (ATP) plays an important role as a primary molecule for the transfer of chemical energy to drive biological processes. ATP also functions as an extracellular signaling molecule in a diverse array of eukaryotic taxa in a conserved process known as purinergic signaling. Given the important roles of extracellular ATP in cell signaling, we sought to comprehensively elucidate the pathways and mechanisms governing ATP efflux from eukaryotic cells. Here, we present results of a genomic analysis of ATP efflux from *Saccharomyces cerevisiae* by measuring extracellular ATP levels in cultures of 4609 deletion mutants. This screen revealed key cellular processes that regulate extracellular ATP levels, including mitochondrial translation and vesicle sorting in the late endosome, indicating that ATP production and transport through vesicles are required for efflux. We also observed evidence for altered ATP efflux in strains deleted for genes involved in amino acid signaling, and mitochondrial retrograde signaling. Based on these results, we propose a model in which the retrograde signaling pathway potentiates amino acid signaling to promote mitochondrial respiration. This study advances our understanding of the mechanism of ATP secretion in eukaryotes and implicates TOR complex 1 (TORC1) and nutrient signaling pathways in the regulation of ATP efflux. These results will facilitate analysis of ATP efflux mechanisms in higher eukaryotes.

Adenosine 5′-triphosphate (ATP) is a coenzyme that functions in the transfer of chemical energy in the cell. In addition to its central role in facilitating cellular energetics, ATP is also thought to be one of the most primitive and widespread extracellular signaling molecules in bacterial, fungal, plant, and animal taxa (Burnstock and Verkhratsky 2009). Purinergic signaling by extracellular ATP plays roles in diverse physiological processes including cell proliferation, development, neurotransmission, and motility. In animals, many of these functions are mediated through vesicular ATP secretion and ATP-binding to purinergic receptors on target cells (Burnstock 1996, 2012). Nineteen purinergic receptors have been described in mammals and they regulate processes as varied as neuronal-glial signaling, platelet activation, and insulin secretion (Abbracchio *et al.* 2009; Geisler *et al.* 2013). Robust glucose-dependent ATP efflux has been observed in *Saccharomyces cerevisiae* and has been shown to occur through a vesicular pathway (Boyum and Guidotti 1997; Zhong *et al.* 2003). However, yeast do not appear to have homologs of mammalian purinergic receptors and the biological functions of ATP efflux in *S. cerevisiae* have yet to be established. It has been reported that secretion of purines has a role in synchronizing sporulation among cells in yeast cultures, suggesting a primitive role for purines in cell-cell communication (Jakubowski and Goldman 1988).

A yeast study identified the transmembrane protein Mcd4 as critical for ATP uptake into the secretory pathway (Zhong *et al.* 2003). This work demonstrated that inhibition or mis-sorting of the vacuolar H^+^-ATPase (vATPase) reduces Golgi levels and extracellular efflux of ATP (Zhong *et al.* 2003). These observations support the idea that ATP secretion occurs via vesicle-mediated mechanisms and that uptake of ATP into the secretory pathway happens upstream of the Golgi requiring a proton gradient. These ideas were later confirmed by studies in mammalian cells (Geisler *et al.* 2013). The late endosome is a critical organelle involved in sorting and trafficking proteins between the Golgi, cell membrane, and vacuole (Rieder *et al.* 1996). Over 60 yeast mutants interfering with late endosomal trafficking have been identified and grouped into five classes based upon endosomal morphology. These loss-of-function mutations result in a wide range of cellular consequences including transcription defects, vacuolar dysfunction, and secretion deficiencies (Bowers and Stevens 2005; Zhang *et al.* 2008). These pleiotropic effects demonstrate the broad role played by the late endosome in cellular processes that require vesicle trafficking.

To gain an in-depth understanding of the cellular mechanisms regulating ATP secretion, we performed a genomic screen to identify genes that modify this process in *S. cerevisiae*. In this screen, we measured extracellular ATP levels in stationary cell cultures in all strains in a complete yeast deletion library. Because deletion strains in the library have different growth rates, ATP measurements were performed in stationary cultures (30 hr postinoculation) in order to allow all deletion strains to reach comparable cell densities at the time of the assay. Our results identified several cellular components, including genetically intact mitochondria, as critical to maintaining wild-type extracellular ATP levels. We also found vesicle sorting to the late endosome as a significant regulator of extracellular ATP. Additionally, we discovered that the amino acid sensing EGO complex, the TORC1 kinase, and retrograde signaling all had significant effects on extracellular ATP levels. We postulate that this represents a central conserved nutrient-responsive and mitochondrial quality control pathway that regulates ATP production and efflux. Our study represents a significant advance toward understanding the relationship between extracellular ATP and nutrient signaling in yeast. More broadly, our work can be used to further studies aimed at understanding conserved signaling pathways and mechanisms regulating ATP secretion in higher eukaryotes.

## Materials and Methods

### Yeast strains and methods

A S288C homozygous deletion library (Open Biosystems, V1.0) was used for the primary screen and follow-up experiments using specific deletion strains. Retests were compared to the isogenic parent strain; BY4743 (*MATa/*α, *ura3*Δ*0/ura3*Δ*0*, *leu2*Δ*0/leu2*Δ*0*, *his3*Δ*0/his3*Δ*0*, *LYS1/lys1*Δ*0*, *met15*Δ*0/MET15*, *xxx*::*KanMX/xxx*::*KanMX)*. Rapamycin (Sigma) stocks were dissolved in DMSO and diluted to final concentration in growth media.

### Primary ATP efflux screen and data analysis

The deletion library was replicated by inoculation into 96-well plates using a BioMek liquid-handling robot (Perkin Elmer) and grown in 150 μl YPD media (1% yeast extract, 2% bacto peptone, 2% glucose) to saturation at 30° for 48 hr. For the ATP assay, yeast cultures from each plate were replicated by 50-fold dilution into three fresh media plates resulting in three biological replicates of each deletion strain. Assay plates were grown in synthetic complete media (0.67% yeast nitrogen base with ammonium sulfate, 0.156% amino acids, 2% glucose) at 30° for 30 hr. Optical density at 660 nm (OD_660_) was measured from a 1:10 dilution in water in 96-well plates using a VictorX3 plate reader (Perkin Elmer). Additional control plates carrying appropriately diluted medium were measured as blanks (n = 8). Extracellular ATP levels present in each culture well were determined using the ATPlite assay (Perkin Elmer) according to the manufacturer’s instructions. Briefly, the cultures in the undiluted assay plates were cleared by centrifugation and 50 μl of supernatant, 50 μl of mammalian lysis buffer, and 50 μl ATPlite-substrate were mixed and shaken at 750 rpm for 5 min. Luminescence (LUM) of each well was read at 420 nm in a VictorX3 plate reader.

Optical density (OD_660_) values outside of the experimentally derived linear range of the plate reader were excluded from subsequent analysis. Well-specific background OD_660_ absorbance readings were determined by individually averaging values generated in each well among the control plates. These well-specific OD_660_ values were then subtracted from the relative OD_660_ readings based on well position. LUM readings were then divided by background-subtracted OD_660_ values to determine the relative luminescence units per cell (RLU), which are reported as a log2 values. RLU measurements were normalized for plate effects by subtracting the mean of the log2 RLU ratios over all plates for the same well.

### Retesting of selected mutants

Selected strains were streaked from the homozygous deletion library onto YPD plates. Isogenic parent strain and selected mutant strains were inoculated from single colonies into 200 μl liquid cultures of synthetic complete media in a 96-well plate (n = 6), grown for 48 hr, and diluted 50-fold to an assay plate. Assay plates were grown for 30 hr and extracellular ATP was measured as described above. Background LUM and OD_660_ values were subtracted from relevant measurements and retest data are reported relative to values for the parent strain (BY4743). For rapamycin studies, assay plates with parent strain cultures were grown for 26 hr, treated with rapamycin (Sigma) dissolved in DMSO or an equal volume of DMSO alone for 4 hr, and assayed as described. For time-course studies, six biological replicates of each strain were grown and assayed over a 96-hr period for ATP levels as described. Cells inoculated immediately before assay (‘0 hr’), were used to derive assay background levels. Calculated ATP levels are reported relative to background and a student’s *t*-test determined the significance of differences within each time-point compared to the parent strain.

### Gene ontology analysis

Enrichment of gene ontology (GO) terms for strains with extreme levels of extracellular ATP (top and bottom 5%) was analyzed using the *GO Term Finder (v.0.83)* tool available through the *Saccharomyces Genome Database* website, http://www.yeastgenome.org/cgi-bin/GO/goTermFinder.pl (Dwight *et al.* 2002). GO analysis used all genes present in the deletion library as a background, a 0.01 p-value cutoff, and default settings. Enriched terms were parsed for redundancy using GO trimmer (Jantzen *et al.* 2011) and remaining significant terms with a >2-fold enrichment are reported.

### Network analysis and visualization

Strains with extreme levels of extracellular ATP (top and bottom 5%) were anchored to the full functional interaction network YeastNet v.2 (http://www.inetbio.org/yeastnet/). YeastNet is comprised of 5483 proteins and 102,803 interactions derived from gene coexpression, genetic and physical protein interactions, literature and comparative genomics methods (Lee *et al.* 2007). Nodes of the two groups (High and Low ATP) were extracted from the full network into two subnetworks with only their direct interactions. Only those networks with >5 nodes are shown. GO annotations and respiratory mutants were included as node attributes.

## Results and Discussion

### A genomic screen identifies pathways that regulate extracellular ATP levels

**Figure 1:**
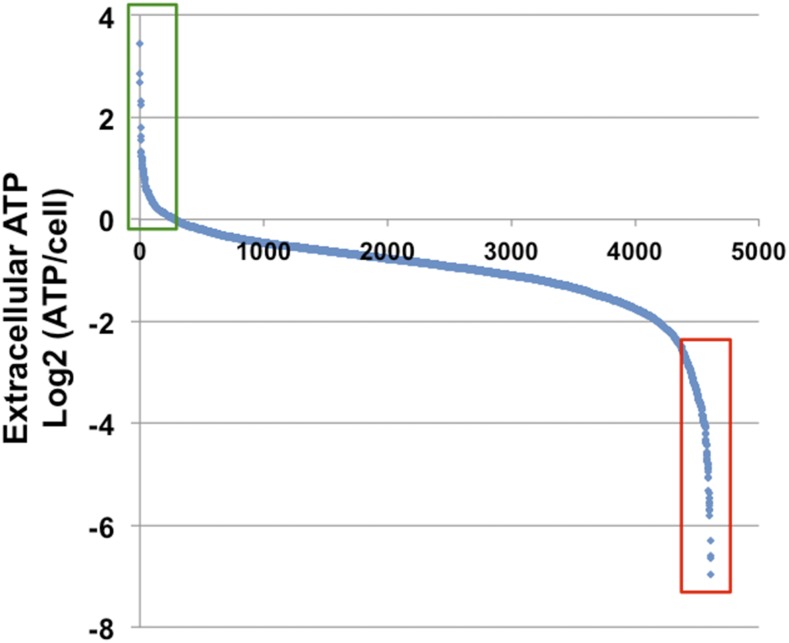
Screen identifying genes regulating ATP efflux. Extracellular adenosine triphosphate (ATP) levels of 4609 unique deletion strains are represented as a Log2 ratio of ATP level/cell. Values shown are the averages of three biological replicates for each deletion strain. Strains are rank ordered from left to right on the x-axis. The 5% of strains (n = 230) exhibiting the highest and lowest levels of extracellular ATP are indicated (green and red rectangles). These two sets constitute the High and Low ATP groups analyzed in this study.

To identify genes that influence ATP efflux from yeast, we measured the extracellular ATP concentration in stationary culture medium for all the 5109 strains in the yeast homozygous deletion library. We reasoned that identifying mutants with particularly high or low levels of extracellular ATP would elucidate the signaling pathways and cellular components that regulate ATP secretion from eukaryotic cells. An initial experiment indicated that extracellular ATP in wild-type (BY4743) yeast grown in complete synthetic media with 2% glucose peaked at a concentration of approximately 1–2 μM by 40 hr postinoculation (data not shown). This ATP concentration is consistent with published observations for yeast grown in glucose (Boyum and Guidotti 1997). The homozygous deletion library was grown in triplicate in synthetic complete media (2% glucose) for 30 hr (± 1 hr) at 30°. Cell densities were measured as optical density at 660 nm and ATP concentrations in the cleared media was measured using a luciferase-based luminescence assay. Strains whose optical densities were below the linear range of our instrument in two of three biological replicates (*i.e.*, slow growing deletions) were excluded from further analysis. After correction for plate position effects, normalized luminescence and OD readings were used to determine the average ratio of luminescence to cell density for each deletion strain. This is henceforth referred to as extracellular ATP levels. Of the 5109 samples in the homozygous deletion collection, we were able to accurately determine the relative extracellular ATP levels for 4609 of the 5109 deletion strains in the library (Figure 1 and Supporting Information, Table S1). To identify pathways that influence extracellular ATP levels, we ranked strains by their extracellular ATP level and selected the upper and lower 5% of the distribution (n = 230) to represent the strains with extreme high and low ATP efflux (Figure 1 and Table S1). These two groups are referred to as High and Low Extracellular ATP groups. We reasoned that identifying cohorts of genes with shared features within each group would identify pathways, complexes, and mechanisms that regulate extracellular ATP levels. It is possible that some cell lysis could have contributed to increased extracellular ATP observed in certain strains. However, this was not investigated further.

To better understand how the genes within the High and Low Extracellular ATP groups are related, each group was queried individually for significantly enriched gene ontology (GO) terms (Table 1). GO analysis was carried out using the *Saccharomyces* Genome Database GO term finder tool and redundant categories with a greater than 2-fold enrichment were parsed with GO Trimming Software (Jantzen *et al.* 2011).

**Table 1:** Gene ontology (GO) enrichment in Low and High ATP groups

**GO**	**GO Term**	**ID**	**Hits**	**Bkg**	**Fold**	**p-Value**
Low ATP Group						
BP	Mitochondrial translational initiation	0070124	5	5	19.9	2.3E-04
BP	Intralumenal vesicle formation	0070676	5	5	19.9	2.3E-04
CC	ESCRT-III complex	0000815	4	4	19.9	1.0E-03
BP	tRNA aminoacylation for mitochondrial protein translation	0070127	6	9	13.3	8.5E-04
BP	tRNA aminoacylation	0043039	9	14	12.8	2.1E-06
BP	Ubiquitin-dependent protein catabolic process via MVB sorting pathway	0043162	12	19	12.6	5.5E-09
MF	Proton-transporting ATP synthase activity, rotational mechanism	0046933	5	8	12.4	2.5E-03
MF	Aminoacyl-tRNA ligase activity	0004812	7	12	11.6	7.8E-05
CC	Mitochondrial large ribosomal subunit	0005762	18	35	10.2	7.5E-13
BP	Mitochondrial translation	0032543	43	85	10.1	1.2E-31
BP	Mitochondrial RNA metabolic process	0000959	9	18	9.9	4.3E-05
CC	Endosome membrane	0010008	11	24	9.1	9.3E-07
CC	Mitochondrial ribosome	0005761	28	62	9.0	1.1E-18
CC	Mitochondrial small ribosomal subunit	0005763	9	23	7.8	1.3E-04
CC	Mitochondrial matrix	0005759	41	124	6.6	8.7E-22
BP	Late endosome to vacuole transport	0045324	10	32	6.2	1.6E-03
BP	Mitochondrion organization	0007005	63	229	5.5	5.3E-29
BP	tRNA metabolic process	0006399	16	81	3.9	1.3E-03
MF	Structural constituent of ribosome	0003735	32	165	3.9	2.2E-09
BP	Negative regulation of transcription from RNA polymerase II promoter	0000122	17	90	3.8	1.2E-03
CC	Ribosomal subunit	0044391	33	178	3.7	3.6E-09
CC	Mitochondrial part	0044429	65	371	3.5	1.2E-18
CC	Endosome	0005768	17	101	3.3	1.4E-03
CC	Mitochondrial inner membrane	0005743	22	144	3.0	3.3E-04
BP	Regulation of transcription from RNA polymerase II promoter	0006357	36	280	2.6	6.8E-05
BP	Gene expression	0010467	105	864	2.4	9.3E-19
CC	Mitochondrion	0005739	101	823	2.4	9.7E-19
CC	Intracellular organelle lumen	0070013	59	482	2.4	2.9E-09
BP	Cellular macromolecule biosynthetic process	0034645	94	832	2.2	9.2E-14
BP	Organelle organization	0006996	90	848	2.1	3.6E-11
BP	RNA metabolic process	0016070	64	650	2.0	1.8E-05
High ATP Group						
CC	GSE complex	0034449	4	5	15.9	5.5E-03
BP	Regulation of biological quality	0065008	32	286	2.2	8.1E-03
CC	Nuclear lumen	0031981	36	326	2.2	6.6E-04
BP	Regulation of cellular macromolecule biosynthetic process	2000112	44	448	2.0	4.9E-03
BP	Regulation of primary metabolic process	0080090	55	561	2.0	2.5E-04

Significantly enriched gene ontology (GO) categories for highest 5% and lowest 5% ATP groups are shown. “Hits” represents number of genes in each dataset (total = 230) with indicated GO ID; “Bkg” represents number of genes in the “background” yeast genome (total = 4609) with indicated GO ID; “Fold” represents fold enrichments of hits within a GO category as compared to chance expectation. ATP, adenosine triphosphate; BP, biological process; CC, cellular component; ESCRT, endosomal sorting complexes required for transport; tRNA, transfer ribonucleic acid; MVB, multivesicular bodies; MF, molecular function; GSE, Gap1 sorting in the endosome.

**Figure 2:**
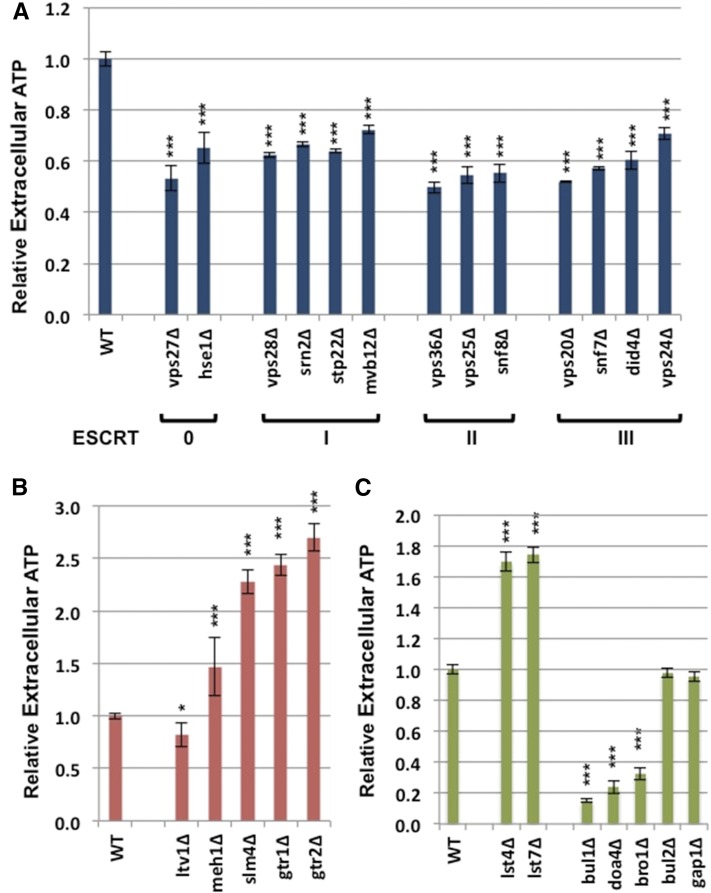
Retest of selected hits identified in the primary screen. Extracellular adenosine triphosphate (ATP) levels are shown for wild-type cells and strains deleted for genes encoding core components of the (A) ESCRT (endosomal sorting complex required for transport) complex, (B) EGO/GSE (exit from G_0_/Gap1 sorting in the endosome) complex, and (C) a group of EGO/GSE interacting proteins. n = 6 biological replicates in all cases. Bars indicate standard error values: *** p < 0.005; * p < 0.05.

Representative deletion strains present as genes in enriched GO categories were subsequently retested using the same growth and assay conditions used in the primary screen. Extracellular ATP levels of all retested deletion strains are shown relative to extracellular ATP levels of the parent control (Figure 2 and Table S2).

GO analyses of genes present in groups indicate that the genes found in the Low Extracellular ATP group share more annotations than those found in the High Extracellular ATP group. 76.1% of the genes in the Low ATP group are included in enriched GO terms as compared to 41.3% included in the High ATP group.

**Figure 3:**
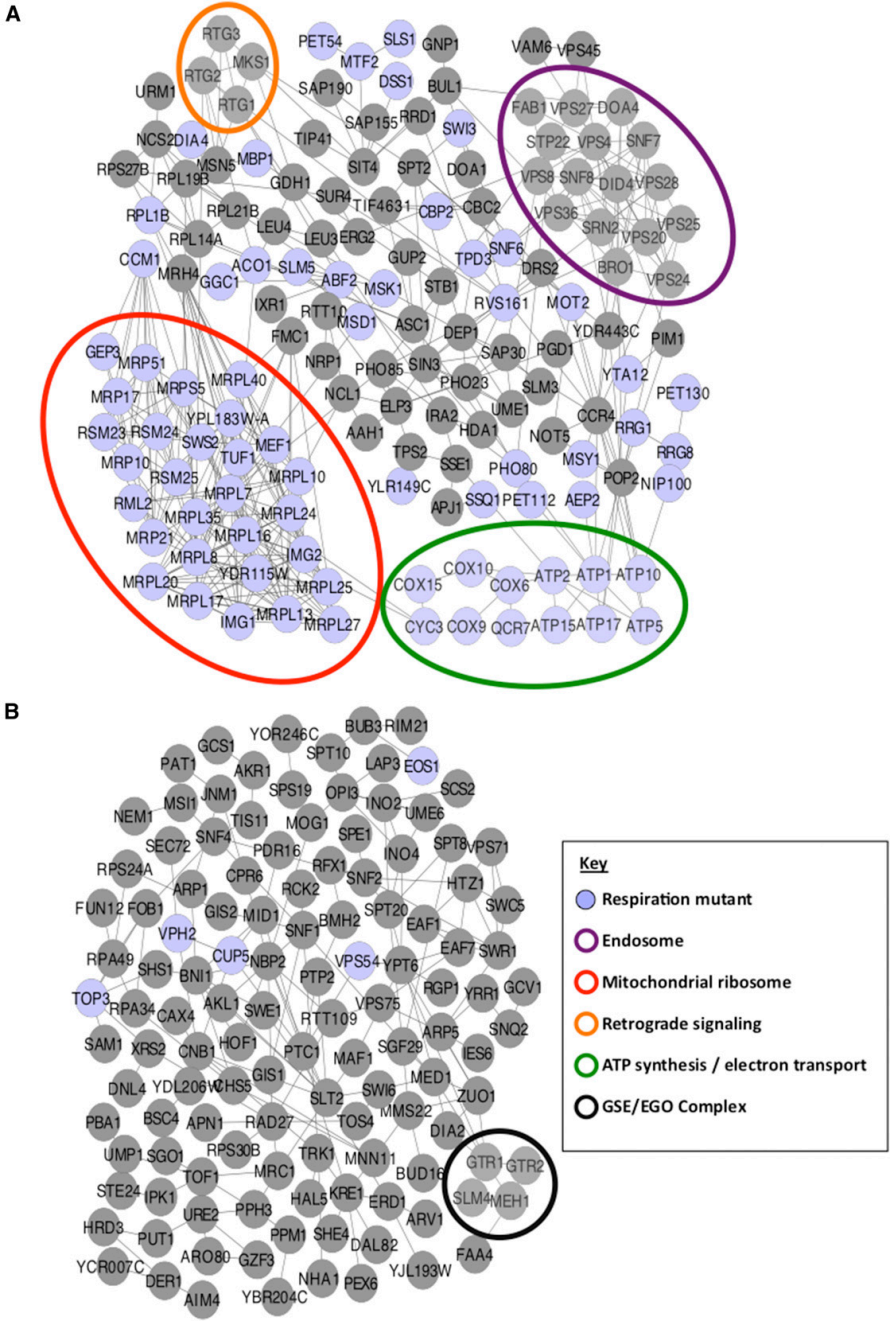
Interaction networks of genes in the High and Low ATP groups. Interaction networks of genes deleted in the High and Low Extracellular adenosine triphosphate (ATP) groups were assembled with data from the YeastNet large-scale interaction network. Relevant modules are shown within the (A) Low Extracellular ATP group (n = 147 nodes) and (B) High Extracellular ATP group (n = 123 nodes). Blue nodes represent respiration-deficient mutants and colored ovals define specific functional subnetworks as indicated in the Key. EGO/GSE, exit from G_0_/Gap1 sorting in the endosome.

To illustrate the functional relationships within these groups, we generated interaction networks for each. This reveals additional relationships between genes that were not captured by GO analysis. Networks were constructed using YeastNet, a probabilistic functional gene network for yeast that incorporates experimental observations from DNA microarrays, physical protein interactions, genetic interactions, literature, and comparative genomics methods (http://www.inetbio.org/yeastnet/). In this analysis, 91.3% and 89.1% of the genes from the Low and High ATP groups, respectively, could be mapped into functional interaction networks (Figure 3) (Lee *et al.* 2007).

These interaction networks illustrate the fact that genes in the Low ATP group are more closely related as compared to those in the High ATP group. The interaction network generated from the Low Extracellular ATP group shows a high degree of connectivity with an overall average of 2.03 edges/node, and comprises a unique module including 70% of the mapped Low ATP genes with only direct interactions, with an average of 2.19 edges/node. This module has two highly connected subnetworks representing components of two distinct cellular machines: the mitochondrial ribosome and the endosomal sorting complexes required for transport (ESCRT) complex (Figure 3A). In contrast, the interaction network generated from the High Extracellular ATP group has an overall connectivity of 0.9 edges/node, comprises a unique module including 60% of mapped genes with an average of 1.48 edges/node, and does not appear to contain any large highly connected subnetworks. We do however observe a subnetwork comprised of four components of the GSE/EGO complex: GTR1, GTR2, SLM4 and MEH1 (Figure 3B). Representative genes present in specific networks modules were retested for ATP efflux as described above (Figure 2 and Table S2).

While the genes that influence extracellular ATP levels represent a wide range of cellular functions, pathways, and processes, GO and protein interaction analyses identified several common features within the datasets. We discuss observations derived from the analysis of the screen and their biological implications below.

### Respiration mutants have decreased extracellular ATP levels

The 5% of strains with the lowest levels of extracellular ATP were enriched for strains with deletions of nuclear-encoded mitochondrial genes. GO categories enriched significantly in the low ATP group included mitochondrial translation, tRNA aminoacylation, ATP Synthesis, and several cellular component categories associated with the mitochondria (Table 1). The functional interaction network for the Low ATP group also illustrates the relationship between deletion of mitochondrial components and low extracellular ATP levels (Figure 3A). Exemplifying this is a highly connected subnetwork of interacting proteins involved in mitochondrial translation (Figure 3A, red oval). Proteins in this subnetwork are primarily mitochondrial ribosomal proteins, suggesting that mitochondrial translation is critical for maintenance of wild-type levels of extracellular ATP. Retest data confirmed that several classes of mitochondrial mutants (representing GO categories: mitochondrial morphology, ATP synthase, mitochondrial ribosomal proteins) have decreased extracellular ATP levels (Table S2).

Mutants with defective mitochondria are known as “respiration deficient” mutants due to their inability to grow on nonfermentable carbon sources (Sherman 1963). Given the enrichment of mitochondrial mutants in our Low ATP group, we reasoned that respiration deficient mutants would also be enriched in this group. This idea was confirmed by comparison of our results with a list of respiration-deficient mutants identified in a screen using a similar homozygous deletion library (Dimmer *et al.* 2002). Our screen measured extracellular ATP levels in 293 of the 341 respiration-deficient strains identified by Dimmer and colleagues (Table S1). Of these, 104 are found in the 5% of strains with the lowest extracellular ATP levels (n = 230), This represents a 7-fold enrichment over what would be expected by chance. By comparison, the High ATP group contains 11 of 230 respiration-deficient mutants. A hyper-geometric test indicates that this is not significantly different than a random distribution (p-value = 0.196).

These results indicate that mutants with compromised mitochondrial function (*i.e.*, respiration-deficient mutants) exhibit decreased extracellular ATP (Table S1). While not unexpected, this indicates that our screen identified physiologically relevant pathways contributing to ATP production. Furthermore, it suggests that extracellular ATP is made primarily via oxidative respiration in the mitochondria, and is likely to be responsive to signals that regulate respiration. Finally, this finding suggests that cell signaling involving mitochondrial function may influence extracellular ATP levels, an idea we explore further below.

### Extracellular ATP levels are regulated by vesicular sorting in the late endosome

Our data indicate that vesicular sorting in the late endosome is critical for the maintenance of extracellular ATP. In both the High and Low extracellular ATP groups, we identified components of machinery that regulate the trafficking of membrane proteins and associated vesicles through the endosome. Notably, components of both the ESCRT (Endosomal Sorting Complexes Required for Transport) and GSE (Gap1 Sorting in the Endosome) complexes were represented among the deletions that exhibited anomalous ATP efflux.

### The ESCRT complex is required for ATP efflux

The GO category “ESCRT-III complex” was highly enriched among the genes denoted the Low ATP group, with all four components being present (Table 1). The ESCRT machinery is comprised of four complexes that regulate the formation of multivesicular bodies (MVBs) in the late endosome. Membrane proteins targeted for degradation are sorted into MVBs and subsequently targeted for degradation in the vacuole. Inhibition of ESCRT function inhibits overall endosomal trafficking and results in an array of cellular phenotypes (Roxrud *et al.* 2010). Analysis of genes in the Low ATP group revealed that mutants of the core components of the ESCRT-0, ESCRT-I, ESCRT-II, and ESCRT-III complexes were all associated with low extracellular ATP. This resulted in additional enrichment of such GO terms as “intralumenal vesicle formation”, “ubiquitin-dependent protein catabolic process via MVB sorting pathway”, “endosome membrane” and “late endosome to vacuole transport” (Table 1). This relationship is also represented in a genetic interaction network as a highly connected subnetwork in the Low ATP group (Figure 3A). Proteins within this subnetwork are not reported to be respiration-deficient suggesting that mitochondrial defects are not driving diminished extracellular ATP in this group. This indicates that the Low ATP phenotype is driven by defects in the ATP efflux rather than ATP supply.

Retesting of strains with deletions in core components of all four ESCRT subcomplexes as well as strains null for ESCRT-interacting proteins confirms that the ESCRT complexes are required for wild-type ATP efflux. Remarkably, deletions in core components of any of the four ESCRT complexes resulted in significantly deceased extracellular ATP levels as compared to the parent strain (between a 1.4–2-fold reduction) (Figure 2A and Table S2). Furthermore, six of the nine ESCRT-interacting protein deletions tested have significantly decreased extracellular ATP levels, indicating that they too are important for ATP efflux. This includes two mutants not scored as having decreased extracellular ATP in the primary screen (Table S2).

This analysis reveals that the ESCRT machinery and, thus, vesicle sorting in the late endosome is critical for ATP efflux. The late endosome is an important organelle for vesicle-mediated endo- and exocytosis and trafficking between intracellular compartments (*e.g.*, cell membrane, Golgi, vacuole). The ESCRT complexes are central to the recycling of proteins via multivesicular bodies (MVBs) that occur at late endosomes, and defects in this process are deleterious to global aspects of cellular vesicular trafficking (Roxrud *et al.* 2010). ESCRT mutants, also known as “Class E vps” mutants, are characterized morphologically by enlarged late endosomes that accumulate cargo targeted for other parts of the cell (Bowers and Stevens 2005; Rieder *et al.* 1996). It would stand to reason that metabolites or proteins that are trafficked through the late endosome might also accumulate in this compartment in ESCRT mutants (Bowers and Stevens 2005). Thus, ESCRT mutants may be affecting ATP secretion by directly sequestering ATP in the late endosome and preventing its vesicle mediated-secretion.

### The GSE complex negatively regulates ATP efflux

Analysis of the strains with the highest levels of extracellular ATP (top 5%) revealed the enriched cellular component GO category “GSE complex” (Table 1). The GSE complex is a five-protein complex that is necessary for sorting of the general amino acid permease Gap1 from the late endosome to the cell membrane upon aminoacid starvation. Although the molecular mechanism is unclear, cells null for GSE complex genes are unable to sort the Gap1 protein from the late endosome to the cell surface in response to amino acid signaling (Gao and Kaiser 2006). Four of the five genes that encode components of the GSE complex were present in the High ATP group (Table S1) suggesting that this complex inhibits ATP efflux.

Retesting confirmed that mutants lacking one of four components of the GSE complex (as observed in the primary screen) have a 1.5–2.7-fold increased extracellular ATP levels than the parent control strain (Figure 2B and Table S2). Mutants carrying a deletion of the gene encoding the remaining 5th component of the GSE complex, *LTV1*, had a 1.2-fold decrease in extracellular ATP compared to the parent (Figure 2B and Table S2). Ltv1-HA has been shown to be the only GSE complex component that does not colocalize with the other components at the late-endosomal membrane, suggesting that Ltv1 complex interaction may be transient or not associated with the function of the complex at the late endosome (Gao and Kaiser 2006). This is also consistent with a specific role for the late endosome in facilitating ATP efflux.

While the GSE complex is critical for promoting Gap1 localization to the cell surface upon amino acid-starvation, the ESCRT complex is important for the recycling of Gap1 from the cell membrane. Thus, our screen negatively correlates biochemical functions that favor trafficking of the Gap1 permease to the plasma membrane with extracellular ATP levels. In addition to the GSE complex, several proteins have been shown to be required for trafficking of Gap1 to the plasma membrane. These include Sec13, Lst4, Lst7, and Lst8 (Roberg *et al.* 1997). Mutations of any of these components prevent Gap1 from trafficking to the cell surface and instead promote its targeting to the vacuole for degradation (Figure S1). The recycling of Gap1 from the plasma membrane to the endosome requires polyubiquitination by the Rsp5/Bul1/Bul2 complex, and efficient sorting within the late endosome requiring Bro1, the deubiquitinase Doa1, and the ESCRT complexes. Mutation of any of these components results in persistent localization of Gap1 at the plasma membrane (Merhi and Andre 2012; Nikko and Andre 2007; Nikko *et al.* 2003) (Figure S1).

To further examine the correlation between Gap1 sorting and extracellular ATP levels, we asked whether other components of the Gap1 sorting pathway can affect ATP efflux. Of the nonessential GSE-sorting components, *lst4∆* and *lst7∆* cultures were present in the High ATP group, while *bul1∆*, *bro1∆*, and *doa4∆* strains were present in the Low ATP group (*SEC13*, *LST8*, and *RSP5* are essential genes and, thus, not present in the deletion library used in the primary screen). Thus, mutants defective in Gap1 sorting to the surface (GSE complex, *LST7*, and *LST4*) have high levels of extracellular ATP, while those deficient in Gap1 internalization (ESCRT complex and interactors, *BUL1*, *BRO1*, *DOA4*) have low levels of extracellular ATP.

Overall, retests confirmed that components promoting Gap1 localization to the plasma membrane resulted in increased ATP efflux, while those that promote Gap1 internalization result in decreased efflux. Both *lst7*∆ and *lst4*∆ cultures had 1.7-fold more extracellular ATP as compared to the parent control (Figure 2C and Table S2). Components involved in Gap1 internalization seemed to have the most drastic effect on ATP efflux. Strains null for *BRO1* or *DOA4* had a 3.1- and 4.2-fold decrease in extracellular ATP as compared to the wild-type, and *bul1∆* cells had the lowest levels of ATP in our retest with a 6.6-fold decrease (Table S2). The *BUL1* paralog *BUL2* did not affect extracellular ATP in either our screen or subsequent retests (Table S1 and Table S2). Finally, retesting of the *gap1∆* strain confirmed that the permease itself does not influence extracellular ATP levels (Figure 2C and Table S2). Together, these observations indicate that the vesicular sorting pathway responsible for trafficking GAP1 has a critical role in ATP secretion.

**Figure 4:**
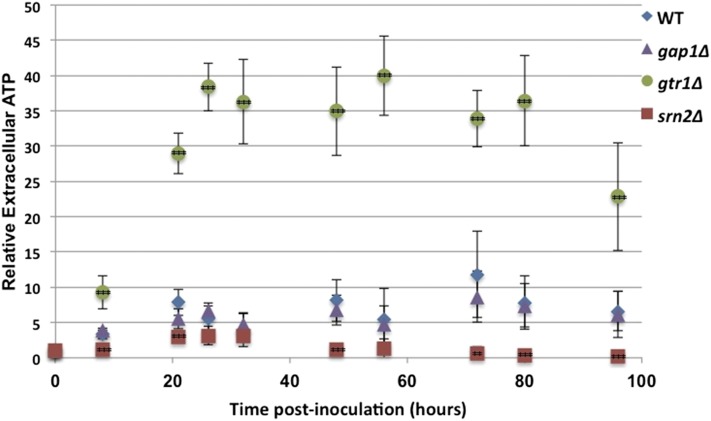
Extracellular ATP levels measured over a 96-hr time-course. The levels of extracellular adenosine triphosphate (ATP) were measured in cultures of wild-type, *gtr1∆* (GSE mutant), *srn2∆* (ESCRT-I mutant), or gap1∆ cells over a 96-hr time-course. Values are normalized to ATP levels at time “0” within each strain. n = 6 biological replicates in all cases. Bars represent standard deviation. *** p < 0.005; ** p < 0.01. ESCRT-I, endosomal sorting complex required for transport I; GSE, Gap1 sorting in the endosome.

Gap1 sorting is responsive to nitrogen availability (Merhi and Andre 2012). We reasoned that nutrient signaling fluctuates during logarithmic growth and entry into stationary phase, and that this might affect ATP efflux. To further characterize the relationship between Gap1 sorting and extracellular ATP in stationary phase, we measured extracellular ATP levels in cultures of wild-type, *gtr1*Δ (a GSE mutant), *srn2*Δ (an ESCRT-I mutant), and *gap1*Δ cells over a 96-hr time-course. Results of this experiment were consistent with observations from the primary screen and subsequent retests. Compared to background levels at the start of the time-course, wild-type cells exhibited a 7.5-fold increase in ATP levels 21 hr after inoculation (Figure 4). Cultures of the GSE mutant *gtr1∆* had significantly higher levels of extracellular ATP than the wild-type control at all time-points tested, supporting the idea that the GSE complex suppresses ATP secretion (Figure 4). *gtr1∆* cultures rapidly accumulated extracellular ATP to 9.2-fold above background within 8 hr of inoculation, and reached over 38-fold by 26 hr. At the same time-point, wild-type cultures had extracellular ATP levels of only 7.1-fold over initial media levels. By comparison, the ESCRT-I mutant *srn2*Δ showed decreased ATP efflux over the entire time-course (Figure 4). ATP levels were slow to rise in cultures of *srn2*Δ cells, peaking at 3.1-fold the initial readings at 26 hr and falling below initial media levels by the 72 hr time-point. As in the primary screen, we observed that *gap1*Δ cultures had similar levels of extracellular ATP as wild-type cultures at all time-points tested (Figure 4), indicating that this specific amino acid permease does not itself modulate extracellular ATP levels.

Our data indicate that cellular processes that regulate Gap1 sorting between the plasma membrane and the endosome also mediate ATP efflux. Those components that promote Gap1 sorting to the cell surface (Lst4, Lst8, GSE complex) negatively regulate extracellular ATP concentrations. Conversely, proteins involved in recycling Gap1 from the surface (*e.g.*, Bul1) and sorting the permease into MVBs for degradation (*e.g.*, Doa4, Bro1, ESCRT complex) promote ATP efflux (Figure S1). Therefore, we conclude that this trafficking pathway is involved in sorting components critical for ATP efflux, and/or ATP itself, between the late endosome and the cell surface in a manner independent of Gap1 function.

### Signal transduction pathways affecting extracellular ATP levels

Based upon our screening results, we hypothesized that ATP efflux is a highly regulated process responsive to nutrient signaling cues and mitochondrial function. Analysis of our screening data reveals two linked signaling pathways with integrated roles in regulating ATP efflux: EGO (Exit from G_0_) and TORC1 (target of rapamycin complex 1). Notably, these pathways are regulated by amino acid availability and mitochondrial function and result in a concerted transcriptional response regulated by the retrograde signaling pathway, which was also identified in our screen as important to maintain wild-type extracellular ATP concentrations (Jazwinski 2013). This suggests an important link between extracellular ATP levels, mitochondrial function, and aminoacid signaling. Below, we discuss how these findings and how these signaling pathways may be critical to extracellular ATP levels.

### The EGO complex regulates ATP efflux

Four of the five proteins that comprise the GSE complex are also components of the EGO complex. These are Meh1, Slm4, Gtr1, and Gtr2. Meh1 and Slm4 are important for stabilization, localization, and tethering the complex to the vacuolar membrane (Zhang *et al.* 2012). Gtr1 and Gtr2 are small GTPases homologous to mammalian RagA/B and RagC/D (respectively) that form a heterodimeric complex that regulates EGO function (Zhang *et al.* 2012). As in mammalian cells, an active EGO complex contains Gtr1 (RagA/B) bound to GTP and Gtr2 (RagC/D) bound to GDP (Figure S2). The active EGO complex binds and activates TORC1. TORC1 is a highly conserved central regulator of cell physiology that couples growth and proliferation to nutrient signaling cues, including intracellular amino acid levels. Under growth conditions where amino acids are not limiting, the active EGO complex binds and activates TORC1 promoting translation, cell growth, and proliferation. When amino acids are limiting, the EGO complex is unable to activate TORC1, leading to inhibition of growth and activation of protein degradation, stress-responses, and nitrogen scavenging pathways (Dann and Thomas 2006). Although the specific mechanism of TORC1 activation by the EGO complex has not been characterized in yeast, this signaling pathway is thought to be central to the cell’s response to changes in intracellular amino acid levels (Bonfils *et al.* 2012).

The key mechanism for regulation of Rag GTPases occurs through interaction with specific GTPase activating proteins (GAPs) and guanine nucleotide exchange factors (GEFs). In yeast, there is evidence that two different GAPs regulate the EGO complex, Iml1 and Lst7. Iml1, which interacts with Npr2 and Npr3 in a complex called the Seh-association complex (SEAC), targets the active EGO complex and promotes Gtr1 GTPase activity, resulting in inactivation of the complex (Figure S2) (Panchaud *et al.* 2013). The function of Lst7 is less well characterized in yeast. The mammalian homolog of Lst7, the Folliculin tumor suppressor (FLCN), promotes GTPase activity of RagC/D, the mammalian Gtr2 homolog (Tsun *et al.* 2013). Although the mechanism of Lst7 activity on Gtr2 has not been described in yeast, genetic evidence suggests that Lst7 and Gtr2 function in the same pathway regulating the EGO complex (Tsun *et al.* 2013). Thus, by promoting Gtr2 GTPase activity, Lst7 would promote formation of an active EGO complex (Figure S2). The GEF Vam6 activates the EGO complex by facilitating the exchange of GDP for GTP on Gtr1 (Binda *et al.* 2009) (Figure S2).

Our screen shows that deletion of any one of the EGO complex components results in increased extracellular ATP levels (Figure 2B and Table S1). Notably, the strain for the one gene unique to the GSE complex (*ltv1∆*) was not identified in our preliminary screen as affecting extracellular ATP levels, and exhibited a modest decrease in extracellular ATP levels in retest experiments (Figure 2B, Table S1, and Table S2). Thus, *gtr1∆*, *gtr2∆*, *meh1∆*, and *slm4∆* mutants might be influencing ATP levels via their activity on TORC1, and not directly via vesicle sorting as described above.

Our initial screen also identified GAPs and GEFs that regulate the activity of the Gtr1 and Gtr2. Strains lacking either *NPR2* or *NPR3*, components of the SEAC with Gtr1 GAP activity, showed low levels of extracellular ATP in the initial screen (Table S1) and the retest showed that cultures of *npr2∆* cells had modest, yet significantly lower extracellular ATP levels than the wild-type control (Table S2). This is consistent with the function of Iml1/Npr2/Npr3 SEAC complex as a GAP for Gtr1 (Figure S2). We found that the putative Gtr2 GAP, Lst7, also affects extracellular ATP levels. In both the primary screen and subsequent retests, the *LST7* deletion strain showed elevated levels of extracellular ATP (Figure 2C, Table S1, and Table S2), supporting a model in which Lst7 GAP activity promotes EGO complex activity via Gtr2 (Figure S2).

Overall, our data indicates that the active EGO complex can suppress ATP efflux. In cells with mutations that stabilize the active complex (*npr2∆* and *npr3∆*), extracellular ATP is decreased. In cells that promote an inactive EGO complex (*lst7∆*, *meh1∆*, *slm4∆*, *gtr1∆*, or *gtr2∆*), extracellular ATP is elevated. Since the active EGO complex binds to and stimulates TORC1 activity (Binda *et al.* 2009), we investigated whether EGO activation suppressed extracellular ATP levels via TORC1.

**Figure 5:**
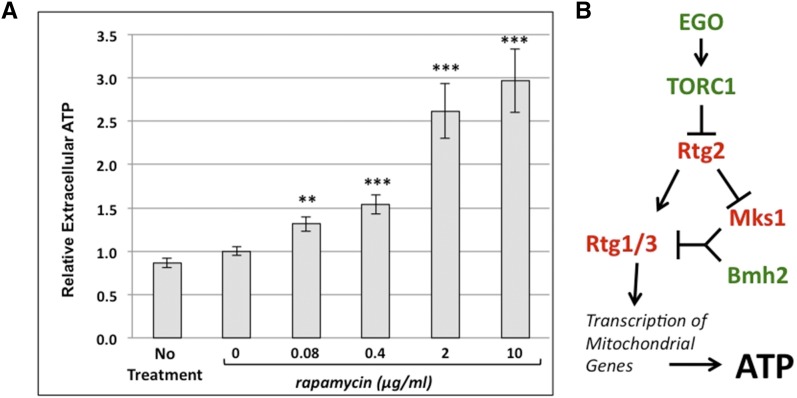
Inhibition of TORC1 by rapamycin enhances ATP efflux. (A) Wild-type cells were grown for 26 hr and treated with vehicle only (0) or with rapamycin at indicated concentrations for 4 hr before extracellular adenosine triphosphate (ATP) was quantified. *** indicates p-value < 0.005; ** indicates p-value < 0.01. (B) Model of interaction between EGO, TORC1, and RTG transcriptional complex function in regulation of ATP production via mitochondrial function. Null mutants encoding proteins/protein complexes shown in green exhibit increased ATP efflux. Those shown in red exhibit decreased ATP efflux. EGO, exit from G_0_; RTG, retrograde; TORC1, TOR complex 1.

To test this, we measured extracellular ATP levels from cultures of wild-type cells that were treated with rapamycin during the last 4 hr of the 30-hr outgrowth. As rapamycin is an inhibitor of TORC1, we reasoned that ATP efflux would be elevated in rapamycin-treated cells. We found a dose dependent increase in extracellular ATP upon treatment with rapamycin. Treatment with 10 µg/ml rapamycin resulted in an approximately 3-fold increase in extracellular ATP as compared to control cultures (Figure 5A). We noted that rapamycin treatment at either 2 or 10 µg/ml elicited similar enhancement of extracellular ATP levels as seen in *gtr1∆* cells. These data are consistent with those showing that the EGO complex regulates ATP efflux via TORC1 (Figure 5B).

### Regulation of ATP efflux by retrograde signaling

The mitochondrial retrograde (RTG) signaling cascade coordinates mitochondrial physiology and nuclear transcription to regulate cellular processes such as energy homeostasis, fatty acid metabolism, and apoptosis. The RTG pathway responds to changes in mitochondrial function and nutrient sensing pathways to modulate transcriptional responses to environmental conditions. Transcriptional activation by the RTG pathway is mediated by Rtg1/3, a heterodimer of two basic helix-loop-helix leucine-zipper proteins. Activation of the RTG pathway results in dephosphorylation and translocation of the Rtg1/3 complex from the cytoplasm to the nucleus (Jazwinski 2013).

Central in the regulation of the Rtg1/3 localization is Rtg2, a sensor of intracellular ATP levels and a transducer of amino acid signaling via TORC1 (Dilova *et al.* 2002; Zhang *et al.* 2013). When TORC1 is active, hyper-phosphorylated Rtg3 is localized in the cytoplasm by phosphorylated Mks1 in complex with Bmh1/2 proteins. When TORC1 is inactive, Rtg2 is thought to dephosphorylate Rtg3 and bind hypo-phosphorylated Mks1 via an ATP-dependent mechanism (Zhang *et al.* 2013). This results in sequestration of Mks1 from the Bmh1/2 complex, facilitating ubiquitination and degradation of Mks1 by the Grr1 SCF-ubiquitin ligase (Sekito *et al.* 2000). Thus Rtg1, Rtg2, Rtg3, and Grr1 are positive regulators of the retrograde signaling pathway while TORC1, Bmh1, Bmh2, and Mks1 are negative regulators of this pathway.

Cells deleted for *RTG1*, *RTG2*, or *RTG3* exhibited low levels of extracellular ATP in the primary screen, suggesting that these genes promote ATP efflux (Table S1). Conversely, *bmh2∆* cells produce high levels of extracellular ATP (Table S1). Taken together, these data suggest that the RTG pathway regulates ATP efflux. Conversely, cultures of *mks1∆* cells had low concentrations of extracellular ATP (Table S1). Retest of components in this pathway showed that cultures of either *rtg1∆* or *rtg3∆* showed a modest but significant decrease in levels of extracellular ATP as compared to wild-type controls (1.4- and 1.3-fold decrease, respectively). Cultures of *rtg2∆* or *mks1∆* cells, however, have extracellular ATP levels similar to the wild-type (Table S2). Thus, while our data directly implicates the retrograde signaling transcription factor Rtg1/3 in the regulation of extracellular ATP, regulation of this phenotype by additional components of the RTG pathway remains to be more fully characterized.

### Interaction between extracellular ATP-regulating components

The results of our screen demonstrate that mitochondrial function, vesicular sorting, EGO-GSE/TORC1 amino acid signaling, and the retrograde response have integrated roles in regulating ATP efflux from *S. cerevisiae*. While we have discussed the relationships between genes found in our screen in the context of discrete mechanisms and pathways, there are considerable associations between them. For example, the role of the retrograde signaling Rtg1/3 transcription factor in promoting ATP efflux is consistent with our findings that inactivation of either EGO or TORC1 inhibition by rapamycin enhance ATP efflux, since the retrograde response is negatively regulated by TORC1 (Figure 5B) (Komeili *et al.* 2000). This association demonstrates interactions between amino acid signaling via the EGO complex and regulation of extracellular ATP, suggesting that these processes are coregulated.

Our data also indicate that functional mitochondria are required for high levels of extracellular ATP, as respiration-deficient mutants exhibit low efflux. The retrograde signaling pathway activates transcription of many nuclear-encoded mitochondrial genes, including those encoding components of the tricarboxylic acid cycle (TCA) (Hashim *et al.* 2014). Thus, activation of the retrograde response promotes increased respiration and, in turn, ATP production. A simple model representing the interaction between these findings predicts that the EGO/TORC1 complex regulates the RTG transcriptional response, which in turn promotes ATP production in the mitochondria (Figure 5B). While aspects of this pathway have been reported in other studies (Dilova *et al.* 2002; Dubouloz *et al.* 2005; Komeili *et al.* 2000; Zhang *et al.* 2013), the effect that this pathway has upon ATP efflux is a novel finding. Supporting this idea, intracellular ATP suppresses the RTG pathway by inactivating Rtg2 and promoting suppression of the Rtg1/3 transcription factor by Mks1 (Zhang *et al.* 2013). Furthermore, RTG pathway activation also promotes glutamate production in the mitochondria, which in turn can activate TORC1 (Dubouloz *et al.* 2005). Both these mechanisms represent feedback loops regulating the EGO/TORC1/RTG pathways that appear to regulate extracellular ATP levels (Figure 5B).

Maintaining intracellular ATP homeostasis is critical for proper cellular function (Walther *et al.* 2010), and ATP efflux may also correlate with other physiological events that promote ATP production. Supporting this idea, wild-type cells have a drastic increase in ATP during the diauxic shift when cells switch from fermentation (with relatively lower ATP production) to oxidative phosphorylation (with relatively higher ATP production) (Figure 4). Mutations that enhance mitochondrial respiration via RTG signaling might also promote ATP production. Retrograde signaling activates the transcription of genes involved in the glyoxylate cycle, acetyl-CoA production, and the mitochondrial import of metabolites for amino acid synthesis (Butow and Avadhani 2004; Hashim *et al.* 2014). By activating the TCA cycle in cells that do not have mitochondrial defects, ATP synthesis is likely increased. Therefore, in conditions that deregulate RTG signaling suppression (*i.e.*, EGO mutants, TORC1 inhibition), extracellular ATP may be elevated as a result of increased ATP synthesis.

This study represents a critical step in identifying the cellular pathways and molecular mechanisms that regulate ATP efflux. Taken together, our screen has identified a central signaling pathway, responsive to both amino acid levels and mitochondrial function, that regulates extracellular ATP levels (Komeili *et al.* 2000). Furthermore, we report that vesicular sorting at the late endosome is also critical for extracellular ATP efflux. These results provide a basis for examining broader questions about the function and regulation of ATP efflux in yeast. It will also be of interest to determine whether and to what degree mechanisms regulating ATP efflux in yeast are conserved in mammalian systems in the contexts of metabolism, nutrient sensing, and purinergic signaling.

## References

[bib1] Abbracchio M. P. Burnstock G. Verkhratsky A. Zimmermann H., 2009 Purinergic signalling in the nervous system: an overview. Trends Neurosci. 32: 19–29. 19008000 10.1016/j.tins.2008.10.001

[bib2] Binda M. Peli-Gulli M. P. Bonfils G. Panchaud N. Urban J., 2009 The Vam6 GEF controls TORC1 by activating the EGO complex. Mol. Cell 35: 563–573. 19748353 10.1016/j.molcel.2009.06.033

[bib3] Bonfils G. Jaquenoud M. Bontron S. Ostrowicz C. Ungermann C., 2012 Leucyl-tRNA synthetase controls TORC1 via the EGO complex. Mol. Cell 46: 105–110. 22424774 10.1016/j.molcel.2012.02.009

[bib4] Bowers K. Stevens T. H., 2005 Protein transport from the late Golgi to the vacuole in the yeast Saccharomyces cerevisiae. Biochim. Biophys. Acta 1744: 438–454. 15913810 10.1016/j.bbamcr.2005.04.004

[bib5] Boyum R. Guidotti G., 1997 Glucose-dependent, cAMP-mediated ATP efflux from Saccharomyces cerevisiae. Microbiology 143(Pt 6): 1901–1908. 9202466 10.1099/00221287-143-6-1901

[bib6] Burnstock G., 1996 P2 purinoceptors: historical perspective and classification. Ciba Found. Symp. 198: 1–28, discussion 29–34. 8879816 10.1002/9780470514900.ch1

[bib7] Burnstock G., 2012 Purinergic signalling: Its unpopular beginning, its acceptance and its exciting future. BioEssays 34: 218–225. 22237698 10.1002/bies.201100130

[bib8] Burnstock G. Verkhratsky A., 2009 Evolutionary origins of the purinergic signalling system. Acta Physiol. (Oxf.) 195: 415–447. 19222398 10.1111/j.1748-1716.2009.01957.x

[bib9] Butow R. A. Avadhani N. G., 2004 Mitochondrial signaling: the retrograde response. Mol. Cell 14: 1–15. 15068799 10.1016/s1097-2765(04)00179-0

[bib10] Dann S. G. Thomas G., 2006 The amino acid sensitive TOR pathway from yeast to mammals. FEBS Lett. 580: 2821–2829. 16684541 10.1016/j.febslet.2006.04.068

[bib11] Dilova I. Chen C. Y. Powers T., 2002 Mks1 in concert with TOR signaling negatively regulates RTG target gene expression in S. cerevisiae. Curr. Biol. 12: 389–395. 11882290 10.1016/s0960-9822(02)00677-2

[bib12] Dimmer K. S. Fritz S. Fuchs F. Messerschmitt M. Weinbach N., 2002 Genetic basis of mitochondrial function and morphology in Saccharomyces cerevisiae. Mol. Biol. Cell 13: 847–853. 11907266 10.1091/mbc.01-12-0588 PMC99603

[bib13] Dubouloz F. Deloche O. Wanke V. Cameroni E. De Virgilio C., 2005 The TOR and EGO protein complexes orchestrate microautophagy in yeast. Mol. Cell 19: 15–26. 15989961 10.1016/j.molcel.2005.05.020

[bib14] Dwight S. S. Harris M. A. Dolinski K. Ball C. A. Binkley G., 2002 Saccharomyces Genome Database (SGD) provides secondary gene annotation using the Gene Ontology (GO). Nucleic Acids Res. 30: 69–72. 11752257 10.1093/nar/30.1.69 PMC99086

[bib15] Gao M. Kaiser C. A., 2006 A conserved GTPase-containing complex is required for intracellular sorting of the general amino-acid permease in yeast. Nat. Cell Biol. 8: 657–667. 16732272 10.1038/ncb1419

[bib16] Geisler J. C. Corbin K. L. Li Q. Feranchak A. P. Nunemaker C. S., 2013 Vesicular nucleotide transporter-mediated ATP release regulates insulin secretion. Endocrinology 154: 675–684. 23254199 10.1210/en.2012-1818 PMC3548185

[bib17] Hashim Z. Mukai Y. Bamba T. Fukusaki E., 2014 Metabolic profiling of retrograde pathway transcription factors rtg1 and rtg3 knockout yeast. Metabolites 4: 580–598. 25007314 10.3390/metabo4030580 PMC4192681

[bib18] Jakubowski H. Goldman E., 1988 Evidence for cooperation between cells during sporulation of the yeast Saccharomyces cerevisiae. Mol. Cell. Biol. 8: 5166–5178. 3072477 10.1128/mcb.8.12.5166-5178.1988 PMC365619

[bib19] Jantzen S. G. Sutherland B. J. Minkley D. R. Koop B. F., 2011 GO Trimming: Systematically reducing redundancy in large Gene Ontology datasets. BMC Res. Notes 4: 267. 21798041 10.1186/1756-0500-4-267 PMC3160396

[bib20] Jazwinski S. M., 2013 The retrograde response: when mitochondrial quality control is not enough. Biochim. Biophys. Acta 1833: 400–409. 22374136 10.1016/j.bbamcr.2012.02.010 PMC3389569

[bib21] Komeili A. Wedaman K. P. O’Shea E. K. Powers T., 2000 Mechanism of metabolic control. Target of rapamycin signaling links nitrogen quality to the activity of the Rtg1 and Rtg3 transcription factors. J. Cell Biol. 151: 863–878. 11076970 10.1083/jcb.151.4.863 PMC2169436

[bib22] Lee I. Li Z. Marcotte E. M., 2007 An improved, bias-reduced probabilistic functional gene network of baker’s yeast, Saccharomyces cerevisiae. PLoS One 2: e988. 17912365 10.1371/journal.pone.0000988 PMC1991590

[bib23] Merhi A. Andre B., 2012 Internal amino acids promote Gap1 permease ubiquitylation via TORC1/Npr1/14–3-3-dependent control of the Bul arrestin-like adaptors. Mol. Cell. Biol. 32: 4510–4522. 22966204 10.1128/MCB.00463-12 PMC3486192

[bib24] Nikko E. Andre B., 2007 Evidence for a direct role of the Doa4 deubiquitinating enzyme in protein sorting into the MVB pathway. Traffic 8: 566–581. 17376168 10.1111/j.1600-0854.2007.00553.x

[bib25] Nikko E. Marini A. M. Andre B., 2003 Permease recycling and ubiquitination status reveal a particular role for Bro1 in the multivesicular body pathway. J. Biol. Chem. 278: 50732–50743. 14523026 10.1074/jbc.M306953200

[bib26] Panchaud N. Peli-Gulli M. P. De Virgilio C., 2013 SEACing the GAP that nEGOCiates TORC1 activation: evolutionary conservation of Rag GTPase regulation. Cell Cycle 12: 2948–2952. 23974112 10.4161/cc.26000 PMC3875668

[bib27] Rieder S. E. Banta L. M. Kohrer K. McCaffery J. M. Emr S. D., 1996 Multilamellar endosome-like compartment accumulates in the yeast vps28 vacuolar protein sorting mutant. Mol. Biol. Cell 7: 985–999. 8817003 10.1091/mbc.7.6.985 PMC275948

[bib28] Roberg K. J. Bickel S. Rowley N. Kaiser C. A., 1997 Control of amino acid permease sorting in the late secretory pathway of Saccharomyces cerevisiae by SEC13, LST4, LST7 and LST8. Genetics 147: 1569–1584. 9409822 10.1093/genetics/147.4.1569 PMC1208332

[bib29] Roxrud, I., H. Stenmark and L. Malerod, 2010 ESCRT & Co. Biology of the cell / under the auspices of the European Cell Biology Organization **102:** 293–318. 10.1042/BC20090161 20222872

[bib30] Sekito T. Thornton J. Butow R. A., 2000 Mitochondria-to-nuclear signaling is regulated by the subcellular localization of the transcription factors Rtg1p and Rtg3p. Mol. Biol. Cell 11: 2103–2115. 10848632 10.1091/mbc.11.6.2103 PMC14906

[bib31] Sherman F., 1963 Respiration-deficient mutants of yeast. I. Genetics. Genetics 48: 375–385. 13977171 10.1093/genetics/48.3.375 PMC1210478

[bib32] Tsun Z. Y. Bar-Peled L. Chantranupong L. Zoncu R. Wang T., 2013 The folliculin tumor suppressor is a GAP for the RagC/D GTPases that signal amino acid levels to mTORC1. Mol. Cell 52: 495–505. 24095279 10.1016/j.molcel.2013.09.016 PMC3867817

[bib33] Walther T. Novo M. Rossger K. Letisse F. Loret M. O., 2010 Control of ATP homeostasis during the respiro-fermentative transition in yeast. Mol. Syst. Biol. 6: 344. 20087341 10.1038/msb.2009.100 PMC2824524

[bib34] Zhang F. Gaur N. A. Hasek J. Kim S. J. Qiu H., 2008 Disrupting vesicular trafficking at the endosome attenuates transcriptional activation by Gcn4. Mol. Cell. Biol. 28: 6796–6818. 18794364 10.1128/MCB.00800-08 PMC2573301

[bib35] Zhang F. Pracheil T. Thornton J. Liu Z., 2013 Adenosine Triphosphate (ATP) Is a Candidate Signaling Molecule in the Mitochondria-to-Nucleus Retrograde Response Pathway. Genes (Basel) 4: 86–100. 24605246 10.3390/genes4010086 PMC3899953

[bib36] Zhang T. Peli-Gulli M. P. Yang H. De Virgilio C. Ding J., 2012 Ego3 functions as a homodimer to mediate the interaction between Gtr1-Gtr2 and Ego1 in the ego complex to activate TORC1. Structure 20: 2151–2160. 23123112 10.1016/j.str.2012.09.019

[bib37] Zhong X. Malhotra R. Guidotti G., 2003 ATP uptake in the Golgi and extracellular release require Mcd4 protein and the vacuolar H+-ATPase. J. Biol. Chem. 278: 33436–33444. 12807869 10.1074/jbc.M305785200

